# Omega-3 Fatty Acids Supplementation in the Treatment of Depression: An Observational Study

**DOI:** 10.3390/jpm13020224

**Published:** 2023-01-27

**Authors:** Seema Mehdi, Kishor Manohar, Atiqulla Shariff, Nabeel Kinattingal, Shahid Ud Din Wani, Sultan Alshehri, Mohammad T. Imam, Faiyaz Shakeel, Kamsagara L. Krishna

**Affiliations:** 1 Department of Pharmacology, JSS College of Pharmacy, JSS Academy of Higher education and Research, Mysuru 570015, India; 2 Department of Psychiatry, JSS Medical College and Hospital, JSS Academy of Higher Education & Research, Mysuru 570015, India; 3 Department of Pharmacy Practice, JSS College of Pharmacy, JSS Academy of Higher Education & Research, Mysuru 570015, India; 4 Department of Pharmaceutical Sciences, School of Applied Science and Technology, University of Kashmir, Srinagar 190006, India; 5 Department of Pharmaceutical Sciences, College of Pharmacy, AlMaarefa University, Ad Diriyah 13713, Saudi Arabia; 6 Department of Clinical Pharmacy, College of Pharmacy, Prince Sattam Bin Abdulaziz University, Al-Kharj 11942, Saudi Arabia; 7 Department of Pharmaceutics, College of Pharmacy, King Saud University, Riyadh 11451, Saudi Arabia

**Keywords:** antidepressants, depression, omega-3 fatty acids, mental health, adjunct therapy

## Abstract

Depression is a common mood disorder characterized by persistent sadness and loss of interest. Research suggests an association between the inclusion of omega-3 fatty acids in the diet and a reduced risk for depression. The present study evaluated the effectiveness of omega-3 fatty acid supplements in alleviating depressive symptoms in patients with mild to moderate depression. A total of 165 patients suffering from mild to moderated depression were randomized to receive omega-3 fatty acid supplementation, an antidepressant (single agent), or a combination of an antidepressant and omega-3 fatty acid supplementation. The clinical features of depression were assessed using the Hamilton Depression Rating Scale (HDRS) during the follow-up period. A statistically significant improvement in depressive symptoms was observed from baseline to first, second and third follow-ups within each treatment arm as measured by HRDS scores (*p* = 0.00001). Further, the HDRS scores at the third follow-up were significantly lower in patients on combination therapy of omega-3 fatty acid supplement and antidepressants (arm-3) than the patients on the omega-3 fatty acid supplement alone (arm-1) [Q = 5.89; *p* = 0.0001] or the patients taking an antidepressant alone (arm 2) [Q = 4.36; *p* = 0.0068]. The combination of an omega-3 fatty acid supplement and an antidepressant elicited significantly higher improvement in depressive symptoms than the supplement or the antidepressant alone.

## 1. Introduction

Depression, also known as major depressive disorder (MDD), is characterized by persistently low or depressed mood, decreased interest, and pleasure in activities, feelings of guilt or worthlessness, lack of energy, poor concentration, appetite changes, psychomotor agitation, sleep disturbances, or suicidal thoughts. According to the World Health Organization, clinical depression is a principal cause of debility worldwide, affecting 5% of the global adult population [1]. The National Mental Health Survey conducted in India between 2015 and 2016 revealed that approximately 15% of Indian adults require external interventions for one or more mental health conditions, with one in 20 individuals suffering from depression [2].

Pharmacological intervention is commonly used to manage clinical depression. A recently conducted drug utilization study in depression patients revealed that escitalopram was the most commonly prescribed antidepressant as monotherapy or in combination [3]. The study also discovered that polypharmacy involving the simultaneous use of two antidepressants was practiced just as much as any agent alone [3]. However, in response to the diminished symptoms or adverse effects associated with these synthetic antidepressants, including withdrawal effects, sexual problems, weight gain, adverse emotional effects, or other factors such as treatment cost, patients tend to discontinue the treatment against medical advice, eventually resulting in the recurrence of episodes [4,5,6]. Hence there is a need to investigate adjunctive or alternative therapy to alleviate symptoms of depression.

Recent reports suggested that, the Hamilton scale for measuring depression symptoms showed a significant improvement when omega-3 fatty acids were added to a typical antidepressant regimen in the majority of patients with treatment-resistant depression, in this regard to improvise the treatment targets a “Neurobiological targeted” techniques, focused on the glutamate system, are being promoted with the objective of encouraging new lines of research aiming at creating therapies that cross the monoaminergic barriers to enhance the treatment of depression disorders [7,8,9]. 

Another study reported that, an add-on therapy of omega-3 has significantly improvized the clinical functioning of sertraline and in addition, Antidepressant effects were amplified by combining antidepressant drugs with dietary and physiological supplements. It is also reported that, when compared to antidepressants alone, adding omega-3 fatty acids in the management of depression was effective and successful with few negative effects [10,11].

Recent studies have demonstrated the usefulness of omega-3, and it has been suggested that taking omega 3 alongside other antidepressants may not be harmful. Another study suggested that combining omega-3 fatty acids with a selective serotonin uptake inhibitor may be beneficial for treating depression at the beginning, and a subsequent experiment found a trend favoring omega-3 supplementation over a placebo in terms of lowering depressed symptoms [12,13,14].

Research suggests that depression can be attributed to several factors, including genetics, abnormal brain chemistry, stressful life events, personality traits, use of certain medications, and due to certain medical conditions [15]. Lifestyle factors, including lack of sleep, exercise, and inadequate diet, also play a significant role. Nutrition plays a vital role in the onset of depression, its severity, and its duration. Studies suggest that nutrition-deficient diets lacking omega-3 fatty acids are associated with higher risks of compromised mental health [16,17]. Omega-3 long-chain polyunsaturated fatty acids (PUFA), including eicosapentaenoic acid (EPA) and docosahexaenoic acid (DHA), are dietary fats found in fish oil are known for their health benefits in fetal development and healthy aging [18]. At the same time, these marine oils are considered vital for brain health because 20% of the brain’s dry weight comprises polyunsaturated fatty acids as a major structural component of neuronal cell membrane phospholipids [19].

Recent evidence suggests that consuming a vegetarian or vegan diet could be linked to an increased risk of depression since vegan diets lack vitamin B_12_, and a vegetarian and vegan diet may be low in long-chain omega-3 PUFAs, as both of these nutrients are critical for the brain health and brain function [20]. The present study evaluates the role of omega-3 fatty acid supplementation in decreasing depressive symptoms in individuals diagnosed with mild or moderate clinical depression and compares its effectiveness with commonly prescribed antidepressants.

## 2. Materials and Methods

### 2.1. Study Design, Setting, and Participants

A prospective observational study was conducted in the psychiatry department’s outpatient unit in a tertiary care hospital in South India for 2 years. A total of 165 patients diagnosed with mild or moderate depression were included in our study after obtaining written consent. In the current study, Hamilton Depression Rating Scale (HDRS) was used to categorize the study participants into either mild or moderate depression. Accordingly, individuals were diagnosed to have mild depression if the total score on 17-item HDRS was between 8 and 13, whereas, moderate depression if the scores were between 14 and 18. 

In this study the patients; (a) with major depression (score ≥ 19) as per HDRS, (b) who were regularly consuming omega-3 fatty acids in any form (c) with any other co-morbid conditions were excluded.

### 2.2. Methodology

This study was approved by the Institutional Review Board of the study site (JSSCPM/IHEC/2019/016). Patients satisfying the study criteria were enrolled and categorized into one of the study arms following a simple randomization technique. Patients in Arm-1 consumed omega-3 fatty acid supplementation (500 mg, daily) but no antidepressants; those in Arm-2 took an antidepressant alone (single agent: Escitalopram, 10 mg, daily or sertraline, 100 mg, daily or fluoxetine, 20 mg, daily) but no omega-3 fatty acid supplementation; and the patients in Arm-3 took an antidepressant (single agent: Escitalopram, 10 mg, daily or sertraline, 100 mg, daily or fluoxetine, 20 mg, daily) along with omega-3 fatty acid supplementation (500 mg, daily). 

The Hamilton Depression Rating Scale (HDRS) was used to estimate baseline and periodic (once a month for three months) changes in clinical features of depression in study participants. Medication adherence to the prescribed antidepressant medications and omega-3 fatty acid supplements were assessed at first, second, and third follow-ups in all three study arms with the help of the Medication Adherence Rating Scale (MARS) score.

### 2.3. Sample Size Calculation

The sample size was calculated to estimate the total number of patients required to compare HRDS scores between three treatment arms using the ANOVA test. Accordingly, the sample size estimations suggested that a minimum of 158 patients were needed to compare the HDRS scores in the different study arms to provide a medium effect, with an effect size of 0.25, power of study of 0.8, and significance level of 0.05.

### 2.4. Statistical Analysis

All the statistical analyses were carried out using Statistical Package for the Social Sciences (SPSS) version 24.0, IBM, Armonk, New York, USA. One-way ANOVA with Tukey HSD was used to assess the differences in the mean HDRS scores among the study participants in the different study arms. A *p*-value of <0.05 was considered statistically significant. 

## 3. Results

The study consisted of 165 patients with mild or moderate depression. All three study arms had an equal number (*n* = 55) of participants. Patient demographics and clinical particulars of study participants are described in Table 1. The mean age of study participants was 37.3 ± 7.9 years; 70.9% of the participants were females. A higher number of participants (52.7%) in the study population had moderate depression.

The MARS scores between the treatment arms did not reveal statistically significant differences (Table 2).

A statistically significant improvement was observed from baseline to first, second and third follow-ups within each treatment arm concerning HRDS scores. There was no significant difference in HDRS scores for the three arms at baseline and first follow-up. However, a statistically significant difference (f-ratio = 3.7728, *p* = 0.0250) was observed between the arms at the second and third follow-ups. The details are presented in Table 3.

The Tukey’s HSD test revealed a significant improvement in HDRS scores in patients who were on combination therapy of omega-3 fatty acid supplement and antidepressants (arm-3) in comparison to patients on the omega-3 fatty acid supplement alone (arm-1) [Q = 3.83; *p* = 0.0203] at the second follow-up. Similarly, a significant improvement in HDRS scores was observed in patients who were on combination therapy of omega-3 fatty acid supplement and antidepressants (arm-3) in comparison to patients who were on the omega-3 fatty acid supplement alone (arm-1) (Q = 5.89; *p* = 0.0001) and the patients who were taking antidepressants alone (arm 2) (Q = 4.36; *p* = 0.0068) at third follow-up. The details are presented in Table 4.

To check the confounding effect of different antidepressants on mean HRDS scores within the treatment arms 2 and 3, the mean HRDS scores among patients on different antidepressants in arm-2 and arm-3 at baseline, first, second and third follow-ups were compared. There was no significant difference concerning mean HRDS scores between the three-antidepressant treatment sub-groups in treatment arm-2 and arm-3 throughout treatment. The details are presented in Table 5.

## 4. Discussion

In the present study population, more women (71%) than men (29%) had mild to moderate depression, which was in agreement with earlier studies which reported depression is twice as common in women than men [21]. One possible explanation for increased rates of depression in women could be hormonal variations, especially during the menstrual cycle, the postpartum period, and perimenopause, which are all associated with mood changes, and predisposing women to anxiety and depression [21]. Further, the patients in the study population belonged to the middle age group (37.3 ± 7.9 years) and consumed a vegetarian diet. According to a study that identified predictors among middle-aged and older people in Europe, apparent social isolation and self-reported poor health were the most prominent risk factors for depression in this age group [22].

With the growing interest in the benefits of veganism and vegetarianism, researchers have tried to establish if this particular way of life is truly all it claims to be. Devoid of vital nutrients like vitamin B12 and omega-3 fatty acids that are necessary for brain function, vegan diets could be associated with depression. Despite having potential advantages like lower rates of obesity, diabetes, heart disease, and cancer, some studies claim that individuals on vegetarian/vegan diets had a higher risk of depression [23].

The adherence to prescribed medications and omega-3 fatty acid supplements in the study participants was assessed using MARS, a validated tool to identify medication adherence of participants, prepared after combining concise concepts from the Morisky Adherence Questionnaire and the Drug Attitude Inventory into the multidimensional self-reporting questionnaire [24]. The tool considers behaviors toward medication adherence, attitudes toward taking medication, and the adverse effects related to psychotropic medication. An individual may receive a maximum of 10 points; higher points suggest improved medication adherence practices.

The MARS score was 7 and above in all three treatment arms during all three follow-up periods, indicating adequate medication adherence in our study population. In addition, when compared for the patients in different arms at each follow-up, MARS scores revealed no significant difference—indicating no adherence bias concerning treatment outcomes. The treatment outcomes were measured using HRDS scores, a scale that measures the severity of depressive symptoms and categorizes patients based on the score as normal or having mild, moderate, or severe depression [25].

A significant improvement in the depressive symptoms of the study subjects was observed post-therapy, as indicated by a significant decline in the HRDS score from baseline to follow-ups (*p* = 0.00001 for each arm). This illustrated that administered antidepressants (fluoxetine, escitalopram, or sertraline), and omega-3 fatty acid supplements were efficacious in treating depression alone as well as in combination. Fluoxetine, escitalopram, and sertraline belong to selective serotonin reuptake inhibitors (SSRIs), the antidepressant class considered to be the first line of management in individuals diagnosed with depression due to their better safety and efficacy profile than other antidepressants [25]. Similarly, the effect of omega-3 fatty acid supplementation in alleviating depressive symptoms can be associated with many published studies—Ryukyus Child Health Study elucidated the inverse association between fish intake and depression; eating less seafood was associated with reduced low omega-3 intake and higher rates of depression during pregnancy [26,27]. Similarly, another investigation into bipolar depression revealed that omega-3 fatty acids may be useful when used as a supplement for depressive symptoms in the patient cohort and that EPA-supplemented food could help relieve their depressive symptoms [28]. Further, some physicians emphasized that omega-3 pills in combination with SSRIs could be a better approach for treating depression [29].

Our study illustrated that the maximum decline in HRDS from baseline to follow-ups was observed in the third arm, where omega-3 supplements were combined with antidepressants, establishing the superiority over individual therapy. This was confirmed by comparing inter-arm HRDS scores, which revealed a significant decline during the second and third follow-ups in the patients treated with the combination, compared to the patients administered with any therapy alone. Researchers hypothesize that the benefits of omega-3 fatty acids in depression could be multi-faceted [30]. Omega-3 fatty acids may change cell signaling and the structure of cell membrane fluidity and lipid bilayer elasticity, affecting how proteins function and interact within the membrane. DHA is a major component of brain membrane phospholipids and hence plays a vital role in neurite outgrowth and influences the release of neurotransmitters. Omega-3 also helps modulate signal transduction; omega-3 PUFAs can act as agonist ligands for various G-coupled protein receptors. They are also known to prevent the expression of inflammatory markers and have a role in modulating immune responses. Omega-3 fatty acids affect mitochondrial function, reactive oxygen species homeostasis, cell proliferation, cell viability, and apoptosis. These positive benefits reflect directly on the neurotransmitter system resulting in better neural and synaptic plasticity leading to decreased neuro-degeneration, inflammation, and oxidative stress [30]. 

Finally, the assessment of HRDS scores within arms 2 and 3 to evaluate the effect of the type of administered antidepressant revealed no statistical difference, confirming that the type of antidepressant administered did not influence the treatment outcome. The results were aligned with other clinical studies which established similar effectiveness of these SSRIs for depressive symptoms [31,32].

There were few reports that previously demonstrated no efficacy of omega-3 fatty acid in the treatment of Major Depression [33], hence the current study focused on mild to moderate depression and found that the combination therapy has ameliorated the symptoms of depression. 

## 5. Conclusions

Omega-3 fatty acids, when combined with three different types of SSRI, resulted in a significant decline in the severity of depressive symptoms as assessed using HRDS scores, irrespective of the antidepressant used. Our study findings support the use of omega-3 fatty acids as an adjunct therapy along with antidepressants to reduce the severity of depression.

## Figures and Tables

**Table 1:** Demographic and clinical details of study participants.

Demographic Details and Therapeutic Intervention	Arm-1 (*n* = 55)	Arm-2 (*n* = 55)	Arm-3 (*n* = 55)	Overall (N = 165)
Age in years				
Mean Age ± SD	38.2 ± 7.7	35.0 ± 8.7	38.7 ± 6.8	37.3 ± 7.9
Gender				
Female	39 (23.6)	37 (22.4)	41 (24.8)	117 (70.9)
Male	16 (9.6)	18 (10.9)	14 (8.4)	48 (29.0)
Diagnosis				
Mild depression	28 (16.9)	25 (15.1)	25 (15.1)	78 (47.2)
Moderate depression	27 (16.3)	30 (18.1)	30 (18.1)	87 (52.7)
Treatment options				
Omega-3 fatty acid supplements, 500 mg daily.	55 (33.3)	NA	55 (33.3)	110 (66.6)
Escitalopram, 10 mg, daily	NA	18 (10.9)	21 (12.7)	39 (23.6)
Sertraline, 100 mg, daily	NA	15 (9.0)	11 (6.6)	26 (15.7)
Fluoxetine, 20 mg, daily	NA	22 (13.3)	23 (13.9)	45 (27.2)

**Table 2:** MARS scores of different treatment arms with ANOVA test.

Follow-Up	MARS Scores (Mean ± SD)			f-Ratio	*p*-Value †
	Arm-1	Arm-2	Arm-3		
**First**	7.52 ± 1.01	7.30 ± 0.83	7.38 ± 0.82	0.8430	0.4322
**Second**	8.61 ± 0.84	8.54 ± 0.95	8.85 ± 0.80	1.8848	0.1551
**Third**	9.12 ± 0.84	9.03 ± 0.79	9.18 ± 0.84	0.4364	0.6470

† *p* > 0.05 illustrated no statistically significant difference between the three arms.

**Table 3:** Comparison of HRDS scores in different treatment arms from baseline to first, second and third follow-ups (ANOVA).

Follow-Up	HRDS Scores (Mean ± SD)			Between the Treatment Arms	
	Arm-1	Arm-2	Arm-3	f-Ratio	*p*-Value
Baseline	13.74 ± 2.29	14.25 ± 2.14	14.41 ± 2.14	1.4019	0.2490
First	13.18 ± 2.09	12.90 ± 2.19	12.67 ± 2.03	0.8022	0.4500
Second	11.89 ± 1.85	11.32 ± 1.75	11.01 ± 1.43	3.7728	0.0250 *
Third	11.12 ± 1.80	10.78 ± 1.78	9.8 ± 1.39	9.3408	0.0001 *
Within the treatment arms:					
f-ratio	19.1728	34.8107	69.5286	--	--
*p*-value	0.00001 *	0.00001 *	0.00001 *	--	--

* *p*-Value < 0.05 is statistically significant.

**Table 4:** Pairwise comparison of HRDS scores between the treatment arms from baseline to first, second and third follow-ups (Tukey’s HSD).

Arms	Mean Scores	Q Value	*p*-Value
Baseline			
Arm-1: Arm-2	Arm-1 = 13.75, Arm-2 = 14.25	1.72	0.4462
Arm-1: Arm-3	Arm-1 = 13.75, Arm-3 = 14.42	2.27	0.2462
Arm-2: Arm-3	Arm-2 = 14.25, Arm-3 = 14.42	0.55	0.9193
First follow-up			
Arm-1: Arm-2	Arm-1 = 13.18, Arm-2 = 12.91	0.96	0.7766
Arm-1: Arm-3	Arm-1 = 13.18, Arm-3 = 12.67	1.79	0.4166
Arm-2: Arm-3	Arm-2 = 12.91, Arm-3 = 12.67	0.83	0.8269
Second follow-up			
Arm-1: Arm-2	Arm-1 = 11.89, Arm-2 = 11.33	2.47	0.1902
Arm-1: Arm-3	Arm-1 = 11.89, Arm-3 = 11.02	3.83	0.0203 *
Arm-2: Arm-3	Arm-2 = 11.33, Arm-3 = 11.02	1.36	0.6035
Third follow-up			
Arm-1: Arm-2	Arm-1 = 11.13, Arm-2 = 10.78	1.53	0.5251
Arm-1: Arm-3	Arm-1 = 11.13, Arm-3 = 9.80	5.89	0.0001 *
Arm-2: Arm-3	Arm-2 = 10.78, Arm-3 = 9.80	4.36	0.0068 *

* *p*-Value < 0.05 is statistically significant.

**Table 5:** Comparison of HRDS scores in patients using different antidepressants in treatment arm-2 and arm-3 from baseline to first, second and third follow-ups (ANOVA).

Treatment Arm-2					
Follow-up	HRDS Scores (Mean ± SD)			F-Ratio	*p*-Value
	Fluoxetine (*n* = 22)	Escitalopram (*n* = 18)	Sertraline (*n* = 15)		
Baseline	14.40 ± 1.94	14.50 ± 2.17	13.73 ± 2.43	0.6088	0.5477
First	13.09 ± 2.13	13.11 ± 2.11	12.4 ± 2.44	0.5450	0.5831
Second	11.22 ± 2.04	11.44 ± 1.85	11.33 ± 1.17	0.0734	0.9293
Third	11.00 ± 1.92	10.55 ± 1.42	10.73 ± 2.01	0.3076	0.7365
Treatment arm-3					
Follow-up	HRDS scores (Mean ± SD)			f-ratio	*p*-value †
	Fluoxetine (*n* = 23)	Escitalopram (*n* = 21)	Sertraline (*n* = 11)		
Baseline	14.34 ± 1.99	14.42 ± 2.06	14.54 ± 2.77	0.0307	0.9697
First	12.26 ± 2.24	13.19 ± 1.40	12.54 ± 2.54	1.1773	0.3161
Second	10.69 ± 1.66	11.19 ± 1.03	11.36 ± 1.56	1.0552	0.3554
Third	9.47 ± 1.59	9.95 ±1.16	10.18 ± 1.32	1.1589	0.3217

† There was no statistically significant difference observed between the sub-groups.

## Data Availability

The data presented in this study are available on request from the corresponding author.
